# Health Effects of a Mixture of Indoor Air Volatile Organics, Their Ozone Oxidation Products, and Stress

**DOI:** 10.1289/ehp.8132

**Published:** 2005-07-21

**Authors:** Nancy Fiedler, Robert Laumbach, Kathie Kelly-McNeil, Paul Lioy, Zhi-Hua Fan, Junfeng Zhang, John Ottenweller, Pamela Ohman-Strickland, Howard Kipen

**Affiliations:** 1 Department of Environmental and Occupational Medicine, University of Medicine and Dentistry of New Jersey–Robert Wood Johnson Medical School, Piscataway, New Jersey, USA; 2 Environmental and Occupational Health Sciences Institute, Piscataway, New Jersey, USA; 3 University of Medicine and Dentistry of New Jersey–School of Public Health, Piscataway, New Jersey, USA; 4 Veterans Affairs Medical Center, East Orange, New Jersey, USA

**Keywords:** building-related illness, lung function, neurobehavioral, ozone, stress, symptoms, volatile organic compounds

## Abstract

In our present study we tested the health effects among women of controlled exposures to volatile organic compounds (VOCs), with and without ozone (O_3_), and psychological stress. Each subject was exposed to the following three conditions at 1-week intervals (within-subject factor): VOCs (26 mg/m^3^), VOCs + O_3_ (26 mg/m^3^ + 40 ppb), and ambient air with a 1-min spike of VOCs (2.5 mg/m^3^). As a between-subjects factor, half the subjects were randomly assigned to perform a stressor. Subjects were 130 healthy women (mean age, 27.2 years; mean education, 15.2 years). Health effects measured before, during, and after each 140-min exposure included symptoms, neurobehavioral performance, salivary cortisol, and lung function. Mixing VOCs with O_3_ was shown to produce irritating compounds including aldehydes, hydrogen peroxide, organic acids, secondary organic aerosols, and ultrafine particles (particulate matter with aerodynamic diameter < 0.1 μm). Exposure to VOCs with and without O_3_ did not result in significant subjective or objective health effects. Psychological stress significantly increased salivary cortisol and symptoms of anxiety regardless of exposure condition. Neither lung function nor neurobehavioral performance was compromised by exposure to VOCs or VOCs + O_3_. Although numerous epidemiologic studies suggest that symptoms are significantly increased among workers in buildings with poor ventilation and mixtures of VOCs, our acute exposure study was not consistent with these epidemiologic findings. Stress appears to be a more significant factor than chemical exposures in affecting some of the health end points measured in our present study.

Between 800,000 and 1.2 million buildings in the United States may be associated with building-related illnesses, and thus, between 30 and 70 million workers are exposed to potentially unhealthy working conditions (Kreiss 1990; Woods 1989). Mixtures of volatile organic compounds (VOCs) and ozone (O_3_) are prominent pollutants in indoor environments (Fan et al. 2003). In some cases, VOCs measured in office buildings are associated with complaints of mucosal irritation and non-specific symptoms such as headache (Hodgson et al. 1991; Ten Brinke et al. 1998). Furthermore, healthy men and women intentionally exposed to similar mixtures of VOCs report increased symptoms of eye, nose, and throat irritation and reduced air quality ratings relative to clean air conditions (Hudnell et al. 1992; Prah et al. 1998). The number of symptoms reported in controlled exposure studies, however, are relatively few and of mild intensity compared with the ongoing complaints of office workers (Apter et al. 1994; Mendell and Smith 1990; Nordstrom et al. 1994; Zweers et al. 1992). Thus, some investigators suggest that when O_3_ reacts with VOCs in building environments, secondary products including ultrafine particles (particulate matter with aerodynamic diameter < 0.1 μm)may mediate the more substantial effects found in offices (Bako-Biro et al. 2004; Fan et al. 2003; Rohr et al. 2002; Weschler and Shields 2000; Wolkoff and Nielsen 2001). Our study assesses a selected suite of subjective and objective markers in response to the following exposure conditions: VOCs, VOCs + O_3_, and ambient air with a 1-min spike of VOCs [masked clean air (MCA)]. We hypothesized that exposure to VOCs or VOCs + O_3_ would result in greater symptom severity, compromised neurobehavioral performance, reduced lung function, and increased salivary cortisol relative to the MCA exposure (hypothesis 1: exposure main effect).

Gender and psychological stress also contribute to health complaints in buildings (Crawford and Bolas 1996; Hodgson 1995; Mendell 1993). For example, Bachmann and Myers (1995) found gender and psychological symptoms to be significant predictors of symptoms in two problem and one nonproblem building. Temperature, uncomfortable humidity, and reported odors, however, were also associated with symptoms in the buildings investigated. Women consistently report the highest prevalence of symptoms (Skov et al. 1989; Stenberg and Wall 1995), although external psychological stress (work load and control) is also associated with complaints (Norback et al. 1990; Ryan and Morrow 1992). Therefore, we chose to include only women in our study and to expose subjects to chemical mixtures with and without psychological stress. We hypothesized that subjects would report significantly greater symptom severity and would show a greater cortisol response when exposed to VOCs or VOCs + O_3_ with psychological stress compared with these exposure conditions without stress or to the MCA condition with or without psychological stress (hypothesis 2: exposure × stress interaction).

In summary, indoor environmental quality is affected by numerous factors, including biological, chemical, and particulate pollutants; temperature and humidity; quality of the heating, ventilation, and air conditioning system; noise; light; and odor (Mendell and Heath 2005). Our present study assessed the interaction of chemical pollutants and psychological stress on subjective (i.e., symptoms) and objective (i.e., cortisol, lung function, neurobehavioral performance) indicators of health effects, while holding temperature, humidity, noise, and light constant. Furthermore, our study added an untested exposure dimension created by combining VOCs with O_3_, shown in previous studies to produce a suite of irritating gas and condensed-phase products (Fan et al. 2003).

## Materials and Methods

### Subjects

One hundred thirty healthy, nonsmoking women, who were on average 27.2 years of age (SD = 8.0) with 15.2 (SD = 1.9) years of education, were recruited via advertisements in local newspapers. The ethnic composition of the sample was as follows: Caucasian, 56% (*n* = 73); black, 10% (*n* = 13); Hispanic, 8% (*n* = 10); Asian, 20% (*n* = 26); other, 6% (*n* = 8). Subjects completed a medical history, physical examination, and standard clinical blood chemistry to rule out previous significant occupational exposure to chemicals and the following health conditions: neurologic disease or brain injury, stroke or cardiovascular disease, serious pulmonary disease including asthma, liver or kidney disease, serious gastrointestinal disorders, known endocrine disease, pregnancy or lactation, and major psychiatric conditions, including psychoses, bipolar disorder, alcoholism or drug abuse, and multiple chemical sensitivity with significant illness behavior or disability. Three hundred forty-one individuals were screened for the study. Fifty-four were excluded at the telephone screening because of medical conditions, 74 never came in for their appointed physical examination, 40 declined to participate after the physical examination, 19 were excluded for medical conditions discovered at the physical examination, 4 dropped out after their first exposure, and 6 dropped out after their second exposure (*n* = 197). Fourteen of the 144 subjects who completed the study were pilot subjects whose data were not included in the analysis.

### Dependent Measures

#### Symptom questionnaire.

Subjects rated each symptom on a ratio scale from 0 (barely detectable/no sensation) to 100 (strongest imaginable) (Green et al. 1996). We chose symptoms based on previous literature assessing the health effects of indoor air mixtures (Molhave et al. 1986; Prah et al. 1998). These symptoms included the cognitive and physical effects expected of VOC mixtures (Hudnell et al. 1992; Molhave et al. 1986), anxiety symptoms associated with the odor of exposure, eye irritation, upper respiratory and lower respiratory symptoms associated with O_3_ and the secondary products generated from the reactions of O_3_ with VOCs (Fan et al. 2003), and somatic symptoms not typically associated with VOC mixtures (Appendix 1) (Dalton et al. 1997).

#### Neurobehavioral.

This computerized divided-attention test of cognitive performance, performance on-line (POL) (Mills et al. 1996) offered five different levels of complexity. The test was validated in alcohol dosing trials and was developed explicitly for use in repeated-measures studies of alcohol and drug effects. POL included a central task in which the subject was presented with two lanes of traffic, divided by a double yellow line. Four conditions of “headlights” and “tail lights” appeared on any one trial. The subject was instructed to press the space bar only when a “safe” condition (i.e., left lane, white headlights, and right lane, red tail lights) existed. The peripheral task required the subject to respond with one of four arrow keys (up, down, left, right) in the direction of the critical stimulus (red octagon among other shapes). Task difficulty increased by increasing the number of distracting stimuli in the peripheral display to a random assortment of different colored circles, squares, and triangles. For the divided-attention display, the subject responded to both central and peripheral critical stimuli. After practice, 10 trials of 45 displays were presented at the most complex level. A composite performance score, composed of seven component scores, including hits, misses, false positives, response latency, and responses to targets at varying levels of visual angle, was the performance variable measured (Badiani et al. 1995).

#### Cortisol assays.

An extensive literature documents the significant (*r* ≥0.90) association between salivary and plasma cortisol (Kirschbaum and Hellhammer 1989, 1994). Salivary flow, which may be affected by anxiety, is not documented to affect the concentration of salivary cortisol (Dirks et al. 1988; Kahn et al. 1988), although circadian rhythm affects cortisol production (Walker et al. 1984). Thus, we tested subjects at the same time of day to control for the well-known circadian effect on cortisol production. Kirschbaum and Hellhammer (1989, 1994) also reported that, although a highly significant correlation is shown between salivary and plasma cortisol, absolute values vary significantly. However, in our study, we evaluated relative change scores rather than absolute values. Because hormonal fluctuations also affect cortisol levels, we measured salivary estradiol at baseline before each exposure session to account for any effects of ovulation on salivary cortisol.

Consistent with Kirschbaum and Helhammer (1989), we collected samples using the Salivette (Sarstedt Inc., Rommelsdorf, Germany) method. We asked the subject to chew on a cotton swab for 60 sec, then place the swab into a Salivette holder and affix the cap. The samples were centrifuged at 3,000 rpm for 10 min, which produced 0.5–1 mL of saliva. The saliva was frozen. Samples were analyzed, blind to exposure condition and subject characteristics, in one of the co-investigators’ laboratories (J.O.). Samples were run in duplicate, and equal numbers of samples from each group were run in the same assays.

#### Pulmonary function test.

With the subjects in standing position with nose clips attached, spirometry was performed using a Multispiro SX spirometry system (Creative Biomedics, Inc., San Clemente, CA) that was calibrated daily. We used the highest value for each parameter from any one of three reproducible tracings before exposure for comparison with the highest value for each parameter postexposure. Forced vital capacity (FVC), forced expiratory volume in 1 sec (FEV_1_), and forced expiratory flow 5–75% (FEF_25–75_) were parameters of interest.

### Independent Variables

#### Public speaking task stressor.

After a 4-min silent preparation period, subjects constructed and delivered a 4-min speech on the following controversial scenarios: *a*) construct a presentation on the causes of gray hair from a *Reader’s Digest* article; *b*) present a position on whether homosexual men should be allowed in the military and be given special civil rights protection; *c*) defend herself against a shoplifting charge in front of three authority figures (al’Absi et al. 1997). In a direct comparison al’Absi et al. (1997) demonstrated that these three different topics caused a similar level of stress (cortisol response). We randomized the order of the three scenarios across subjects such that each subject delivered a speech on a different topic in each of her three exposure conditions. After the preparation period, subjects were told by the experimenter to stand, face the video camera, and begin giving the speech. To enhance the stressfulness of the procedure, we told each subject that the research technician was evaluating the speech as it was given and that three staff members would evaluate a videotape of the speech later; the subject was promised an additional stipend of $10 if she performed well. After completion of the three experimental sessions, all subjects received the added stipend regardless of performance.

#### Controlled environment facility and exposure generation.

The Environmental and Occupational Health Sciences Institute Controlled Environment Facility (CEF) is a stainless steel room 2.2 m × high × 4.1 m wide × 2.7 m deep with a total volume of 25 m^3^. The air supply is treated in a series of conditioning processes, which include air cooling/heating, humidification/dehumidification, and filtration through carbon and high-efficiency particulate air (HEPA) filters. All parameter controls are computer interfaced to maintain constant conditions in the CEF. To simulate a typically ventilated office building and to allow sufficient time for the formation of O_3_–VOC reaction products, the air flow rate through the facility was controlled at 1.8 ± 0.2 air changes/hr for all the exposures conducted in this study. The air supply enters the facility through two diffusers in the ceiling and exits through the perforated stainless steel floor to the exhaust vents. Small brushless fans (to prevent unwanted particle generation from brush degradation) were used in the CEF to ensure that the air was well mixed. During exposure sessions, the relative humidity and temperature were controlled to a range of between 24 and 49% and 73–82°F (23–28°C), respectively. A Teflon partition separated the subjects’ work stations, which were equipped with a computer and typing stand on a stainless steel table. This configuration allowed exposure of two subjects at a time. The technician communicated separately with each subject through headphones and could view subjects at all times through a two-way window. For subject safety, an electrocardiogram (ECG) signal was collected using disposable electrodes and sampled at 996 Hz through the Flexcomp Biomonitoring system 1.51 B (Thought Technology Ltd., Montreal, Quebec, Canada). This allowed continuous monitoring of heart rate during the exposure period.

The composition and relative weight of the VOC mixture used were similar to those in previous indoor air studies (Kjaergaard et al. 1991; Molhave et al. 1986; Otto et al. 1992) and are reported in Table 1 (Fan et al. 2003). *d*-Limonene, the most frequently identified terpene in indoor air, was also added to the mixture. A flask containing the liquid mixture of these 23 compounds was heated to 250°C by a hot plate, flash evaporated, and injected into the clean air stream that delivered the VOC mixture into the CEF. Constant concentrations of chemical compounds can be maintained in the CEF by injection of the chemicals continuously into the air supply, which flows through the CEF without recirculation. The desired concentration of VOCs in the indoor environment was achieved by adjusting the flow rate of the delivering air using a mass flow controller.

Total VOC concentration in the air of the indoor environment was approximately 26 mg/m^3^, with the concentration of each compound below the threshold limit value recommended for occupational exposure by the American Conference of Governmental Industrial Hygienists (1992). MCA was generated by introducing a 1-min spike of the VOC mixture at approximately 10% of the exposure concentration for the VOC exposure condition. The maximum total VOC concentration in the CEF during the MCA condition was approximately 2.5 mg/m^3^. Given the air exchange rate used in the experiments, it took about 90 min for the masking concentration to decay to 0.25 mg/m^3^.

The background O_3_ concentration is < 3 ppb in the CEF. O_3_ generated *in situ* by an O_3_ generator was delivered into the CEF and was maintained at a steady-state concentration of 40 ppb. This was consistent with the intention to examine the effect of products of O_3_–VOC reactions rather than O_3_ itself. Table 2 compares the concentrations of byproducts generated in the VOC + O_3_ condition relative to the VOC condition. Exposure generation and characterization were described in detail in a previous publication (Fan et al. 2003).

### Procedures

Before receiving a complete physical examination, including a medical history review and routine blood chemistries, subjects who met inclusion criteria gave written informed consent according to the University of Medicine and Dentistry of New Jersey–Robert Wood Johnson Medical School Institutional Review Board. To reduce practice and novelty effects, subjects were shown the CEF and trained to perform the following procedures: symptom questionnaire, speech and neurobehavioral tasks, salivary sampling for cortisol and estradiol, nasal lavage, and spirometry. Subjects were randomly assigned to the order of exposure conditions, half with and half without the stressor. Subjects were told during informed consent that they may be asked to perform a public speaking task. They were not told whether they would be performing the task before any exposure session. Thus, subjects were blind to the stress condition. Neither the subject nor the research technicians were told exposure conditions.

Each experimental session was 3 hr in duration and occurred in the morning. Subjects were asked not to use caffeine or alcohol on the day before and on the day of the testing session. Subjects also could not have an active upper respiratory illness (either infection or allergy) or use medication for allergies or other respiratory conditions for 1-week before each exposure session. These conditions were queried by a nurse before participation on each day of exposure, and subjects were rescheduled if necessary.

Two subjects were tested at the same time. On the day of the first exposure session, a pregnancy test was given. Under typical room air conditions, a spirogram was performed in our clinical center before and after each exposure session. Subjects also completed the symptom questionnaire, practiced the POL, and performed baseline nasal lavage and salivary sampling for estradiol analysis in our clinical center.

**Figure 1:**
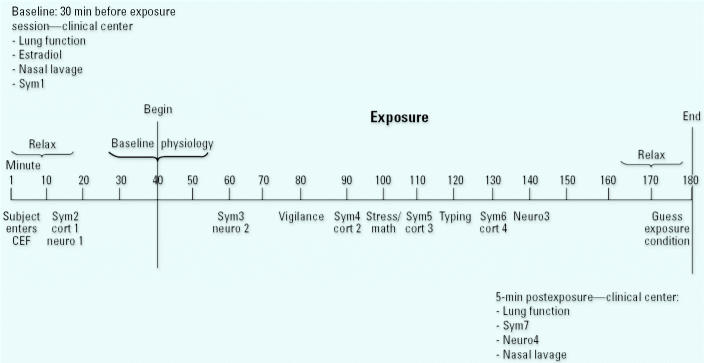
Time line for experimental procedure. Abbreviations: Cort1, cortisol collection at minute 15; Cort2, cortisol collection at minute 90; Cort3, cortisol collection at minute 110; Cort 4, cortisol collection at minute 130; Neuro1, neurobehavioral task (POL) at minute 15; Neuro2, Neurobehavioral task (POL) at minute 60; Neuro3, neurobehavioral task (POL) at minute 140; Neuro4, neurobehavioral task (POL) at 5 min postexposure; Sym1, symptom questionnaire at baseline; Sym2, symptom questionnaire at minute 15; Sym3, symptom questionnaire at minute 60; Sym4, symptom questionnaire at minute 90; Sym5, symptom questionnaire at minute 110; Sym6, symptom questionnaire at minute 130; Sym7, symptom questionnaire at 5 min postexposure.

We escorted subjects to the CEF, where they were seated at a table, had ECG sensors attached, and were given a pair of headphones used to communicate instructions for administration of questionnaires and tasks by the research technician. We gave each subject a loose-leaf binder with dividers to separate each of the questionnaires and tasks to be completed. Baseline measures before exposure were collected while the subject sat quietly with filtered room air, after which the exposure was administered for 140 min (Figure 1). During the initial 20 min of the exposure, subjects read quietly. We administered a 10-min vigilance task to maintain a consistent level of alertness across subjects before introduction of the psychological stressor (Jennings et al. 1992). In this simple color detection task, we instructed subjects to count bars that appeared periodically on a computer screen. After completion of the task, we asked subjects the number of bars counted. Subjects then completed either the public speaking task or simple arithmetic. While subjects performed the simple arithmetic task, they heard classical music through their headphones to screen out the other subject’s voice. After this period, all subjects typed a standard text for 10 min. A different text was used for each experimental session. As indicated in Figure 1, subjects completed questionnaires, collected salivary samples for cortisol analysis, and performed the neurobehavioral task during clean air baseline and the exposure period. Immediately after exposure we escorted subjects to our clinical center, where they performed postexposure spirometry, nasal lavage, the neurobehavioral task, and completed a questionnaires (Figure 1).

### Statistical Analysis

#### Symptoms.

We analyzed the effects of exposure, stress, and time on both presence/absence of symptoms and symptom severity. For presence/absence, if a subject reported any symptom at all, then a “yes” was recorded; otherwise, a “no” was recorded. For presence/absence, a hierarchical logistic regression (Davidian and Giltinan 1995; McCulloch and Searle 2000) modeled the log-odds of no symptoms being reported for assessment at minute 15 (baseline) through minute 185 (after removal from exposure facility). For symptom severity, data were analyzed using a hierarchical Poisson regression model. In both cases generalized estimating equations were used that accounted for correlations between repeated measurements on the same individual (Liang and Zeger 1986). Tests of the exposure effects were conducted using type 3 score tests (Liang and Zeger 1986) of the interaction between exposure and time. Time was entered into the model as a categorical variable. We used contrasts to test whether individual changes in symptoms from baseline (minute 15) to each subsequent time point differed between exposures. The mean odds of reporting symptoms or the mean severity of symptoms at baseline was assumed to be the same for all three exposures. This analysis was first completed for the total symptoms and then for each classification of symptoms: VOC physical, VOC cognitive, eye irritation, anxiety, upper respiratory, lower respiratory, and somatic control symptoms. Results were based on the 130 subjects who received all three exposures, including the MCA condition. Uncorrected α-values are reported, with the αlevel after Bonferroni correction noted for each group of multiple comparisons.

#### Cortisol analyses.

Cortisol is normally elevated during ovulation, potentially attenuating a woman’s cortisol response to a stressor at that time. Therefore, cortisol data were analyzed both with and without data from any specific exposure session in which the subject’s estradiol level was ≥5 ng/mL. Based on this cutoff, 13 of the 130 (10%) subjects were ovulating during their MCA exposure, 13 of 130 (10%) were ovulating during the VOC exposure, and 15 of 130 (11.5%) were ovulating during the VOC + O_3_ exposure. On comparison of the cortisol data analyses with and without ovulation sessions, no differences in outcome were noted. Therefore, sessions in which subjects were ovulating were included in the final analyses of cortisol.

Given the continuous response, SAS PROC MIXED (SAS Institute Inc. 1999) was used to analyze the fixed-factor effects. The cortisol response was right skewed and therefore, for the linear models, was transformed using a square root transformation to better satisfy the normality assumption required for the mixed linear model.

#### Neurobehavioral performance and lung function.

A mixed linear model was used to test the effect of exposure on the composite score from the neurobehavioral task and on indicators of lung function.

## Results

### Hypothesis 1: Exposure Main Effect

#### Symptoms.

**Figure 2:**
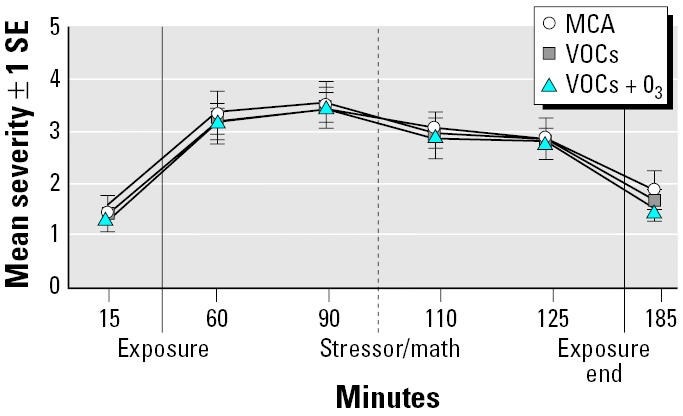
Total mean symptom severity at each time point across exposures.

After controlling for baseline symptoms (minute 15), the overall test of the exposure main effect on total symptom severity did not confirm hypothesis 1 (Figure 2). However, marginal effects for presence/absence of the subscales of VOC physical symptoms (chi squared = 18.01; df = 10; *p* < 0.05) and severity of lower respiratory symptoms (chi squared = 16.92; df = 10; *p* < 0.08) were observed; no effects were observed for the other categories of symptoms.

#### Neurobehavioral performance, salivary cortisol, and lung function.

There was no significant main effect of exposure on neurobehavioral performance (*F* = 1.00, df = 6, 387; *p* = 0.4273), salivary cortisol (*F* = 0.40; df = 6, 316; *p* = 0.888), or lung function (data not shown for neurobehavioral or cortisol measures; see Table 3 for lung function).

### Hypothesis 2: Exposure × Stressor Interaction

#### Symptoms.

**Figure 3:**
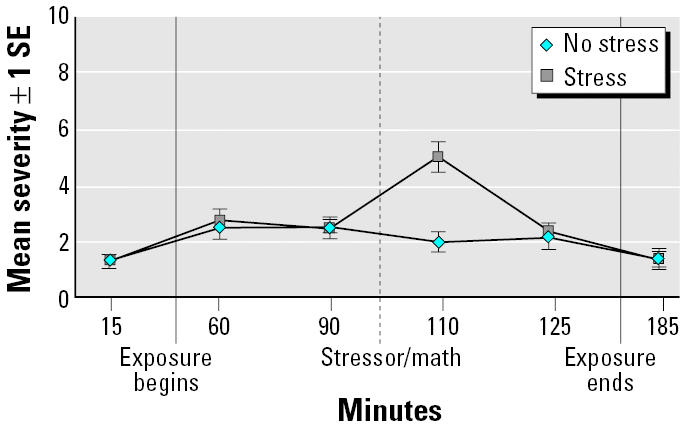
Mean anxiety symptom severity at each time point across exposures stress versus no stress. Bonferroni correction: *p* < 0.003. Time point comparison for anxiety symptom sensitivity is as follows: change from minute 15 (baseline) to minute 110 (poststress), χ^2^ = 15.17 (*p* < 0.0001); change from minute 60 (after exposure onset) to minute 110 (poststress), χ ^2^ = 12.52 (*p* < 0.0004); change from minute 90 (prestress) to minute 110 (poststress), χ ^2^ = 30.16 (*p* < 0.0001); change from minute 110 to minute 125, χ^2^ = 22.29 (*p* < 0.0001); change from minute 110 to minute 185 (poststress), χ ^2^ = 11.64 (*p* < 0.0006).

Hypothesis 2 was not confirmed for presence/absence or for total symptom severity. However, regardless of exposure condition, subjects who were in the stress condition reported significantly greater severity on the anxiety sub-scale (chi squared = 22.73; df = 5; *p* < 0.0004) than those who were not. Specifically, relative to baseline either before (minute 15) or after exposure onset (minutes 60 and 90), symptoms of anxiety were significantly more severe after the stressor at minutes 110 and 125 (Figure 3). No other symptom subscale was significantly affected by stress or by the exposure × stressor interaction.

#### Salivary cortisol.

**Figure 4:**
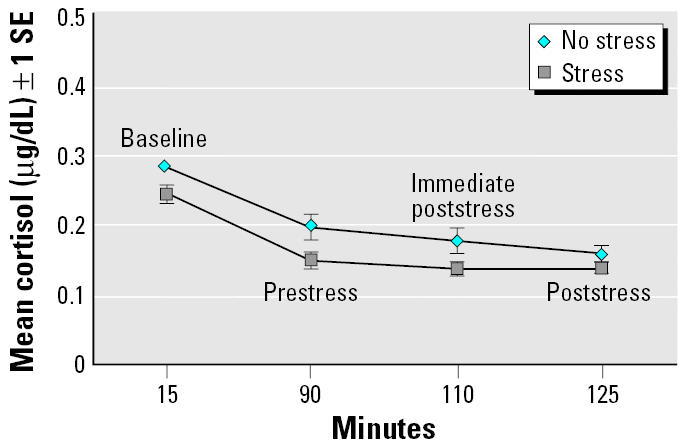
Cortisol over time for stress versus no stress regardless of exposure.

The hypothesized interaction effect of exposure × stress on cortisol was not significant, but the main effect of stress on cortisol was significant (*F* = 4.90; df = 3, 347; *p* = 0.0024). The significance of the main effect of stress was due to changes in cortisol levels from before the stressor (minute 90) to after the stressor (minute 125) (*t* = 4.00; *p* < 0.0001). When examining changes from minutes 110 to 125, on average, the cortisol levels decreased (from 0.178 to 0.154) for the no-stressor condition and increased slightly (from 0.140 to 0.147) for thestressor condition (*t* = 3.63; *p* = 0.0003) (Figure 4).

## Discussion

The most striking result of our study was the lack of significant subjective or objective health effects from exposure to mixtures of VOCs both with and without O_3_, despite significant differences in the chemical composition of the air for the three conditions (Table 2). Numerous epidemiologic studies suggest that symptoms are significantly increased among workers in buildings with poor ventilation and mixtures of VOCs (Mendell 1993; Mendell et al. 2002; Sieber et al. 1996), many of which likely contain low levels of O_3_. Our present controlled acute exposure to similar chemical mixtures in young women, however, failed to support these epidemiologic findings. Relative to ambient air masked with a pulse of VOCs, neither the VOC nor the VOC + O_3_ exposures caused significantly increased symptom reports, changes in cortisol, reduced neurobehavioral performance, or changes in lung function. Subjects were more likely to report some VOC physical symptoms in the VOC and VOC + O_3_ conditions relative to the MCA condition. However, these effects were of marginal significance and became nonsignificant with appropriate correction for multiple comparisons. Conversely, although stress did not exacerbate exposure effects, stress significantly increased symptoms of anxiety. The effect of stress was further validated by the significant difference in cortisol for those who received the stressor relative to subjects who did not.

There was a marginal (*p* < 0.08) increase in severity, but not incidence, of lower respiratory symptoms with the VOC and VOC + O_3_ exposure. However, there were no significant changes in the lung function parameters attributable either to the VOC or VOC + O_3_ exposure. This is consistent with work showing increased lower respiratory symptoms at 50 mg/m^3^ but not at 25 mg/m^3^ of VOCs alone, but no change in spirometry or increase in inflammatory mediators from induced sputum at either VOC concentration, again without O_3_ (Pappas et al. 2000). Although rodent bioassays had indicated that relatively higher concentrations of limonene–O_3_ oxidation products were irritating to the respiratory tract, at our exposure concentrations we did not see an effect on lung function and only a marginal effect on symptoms (Rohr et al. 2002; Wilkins et al. 2001). Indicators of nasal inflammation were also negative for our exposures (Laumbach et al., in press). In contrast to our present findings, Kleno and Wolkoff (2004) reported increases in eye blink frequency among a small number of subjects with eye exposure only to limonene oxidation products and nitrate radicals relative to clean air. Although we did not measure eye blink frequency, symptoms of eye irritation were not significantly greater in the VOC + O_3_ condition.

Our present findings regarding neurobehavioral performance are consistent with those of Otto et al. (1992), who reported no changes in neurobehavioral performance among subjects exposed to a similar mixture of 23 VOCs relative to clean air. However, the research group from the Technical University of Denmark reported several studies in which subjects were exposed to off-gassing from a 20-year-old carpet and showed reductions in productivity on tasks that simulate office work (typing, calculations) (Wargocki et al. 1999, 2000a). Although the total VOCs were of similar concentrations between the exposure conditions with and without the carpet (~ 2.34 ppm), the composition of the chemical mixture differed substantially between the two conditions, and likely contained unidentified compounds associated with emissions from the carpet. In contrast to the negative findings in our present study, Wargocki et al. (1999, 2000a) also reported more symptoms (headache) and slower typing speed in response to the condition with the old carpet (polluted condition). Because the total VOC concentration (2.35 ppm) was less than in our present study, symptom and performance differences could be ascribed to differences in the chemical composition, to unspecified biological components, or to differences in task demands. Neither acetone nor acetic acid was present in the VOC mixture used in our present study (only small amounts were produced from the O_3_ and VOC reactions), but such a specific chemical effect seems unlikely. Wargocki et al. (1999, 2000b) also suggested that simulated work tasks (typing, calculations) performed over a longer period of time (265 min) than in our present study were more sensitive to the effects of poor air quality than are standard neurobehavioral tasks. Thus, although the POL was given for approximately 20 min on two occasions during exposure, this did not require the sustained effort needed to perform continuous typing for 47 min on two occasions as in the Wargocki et al. (1999, 2000b) studies. However, the use of an old carpet as an exposure was ultimately not comparable with the present specific VOC mixture.

Although some increased symptoms were observed in previous controlled exposures using similar “indoor air” mixtures (Hudnell et al. 1992; Prah et al. 1998), actually only a few out of many symptoms assessed in those studies were significantly increased. A careful examination of those symptoms exacerbated by exposure reveals some consistency with our present findings. Prah et al. (1998) reported that relative to clean air, mixtures of VOCs increased ratings of nasal irritation, odor intensity, and air quality but not health (cough, sore throat) and cognitive symptoms (memory loss, dizziness). Similarly comparing responses of a VOC mixture with clean air, Hudnell et al. (1992) reported significantly reduced air quality ratings and increased odor level, but they also reported increased symptoms of headache, eye irritation, drowsiness, and throat irritation. However, Hudnell et al. (1992) did not control for multiple statistical comparisons among the individual tests of 22 symptoms (*p*-value set at < 0.05). Furthermore, no previous indoor air study has “masked” the clean air condition to control for the effects of odor on symptoms. Several studies suggest that when subjects rate air contaminated with various combinations of limonene, O_3_, and office products (paper), they report dissatisfaction with air quality (Knudsen et al. 2002; Tamas et al., in press). However, these studies did not measure health symptoms.

To further clarify our findings, we conducted power calculations to estimate the size of the effect that would be necessary to detect a change between symptoms reported at baseline (minute 15) and either minute 60 or 125. Based on 130 subjects receiving all three exposures, we calculated the minimum actual symptom difference between the VOC and VOC + O_3_ exposures and the MCA exposure that would be needed to attain a power of 90%. In these power calculations, we assumed that the size of the exposure effect, relative to MCA, was the same for VOCs and VOCs + O_3_. To simplify the calculations, we used changes from baseline as the response variable in a repeated-measures analysis of variance. We calculated the variance components for each response and time point from the existing data. To account for multiple testing, the Bonferroni corrected significance level was used corresponding to the level used for testing in our present study.

The results indicate that in all cases, minimum average increases of between 0.6 and 3.0 points in the average score for each category of symptoms would have to exist in order to detect a difference with a power of 90% (Table 4). With symptom severity scored on a scale of 0 to 100, this indicates that very small changes were detectable with the sample size used in our study.

We performed similar power calculations for the main effect of exposure on neurobehavioral performance, cortisol, and lung function. The minimum detectable difference in neurobehavioral performance between the VOC, VOC + O_3_, and MCA conditions for a power of 90% was calculated at 1.30 using a 0.05 significance level. Because of a within-session learning effect, the MCA exposure resulted in a mean increase from baseline (baseline mean = 32.72; SD = 9.1) of 2.10 points on the neurobehavioral task. With 130 subjects we could detect a relative reduction of 1.30 points resulting in 0.80 point improvement for the VOC or VOC + O_3_ exposure conditions (i.e., 2.10 – 1.30 = 0.80). For cortisol, the minimum detectable difference in changes from baseline needed for a power of 90% was 0.066 μg/dL using a 0.05 significance level. The MCA condition resulted in a mean decrease from baseline (the average baseline = 0.2627; SD = 0.1831) of 0.1148 μg/dL. Thus, a decrease of no more than 0.0488 μg/dL in the VOC or VOC + O_3_ conditions was needed to detect a difference. Finally, for lung function, the minimum detectable differences in changes from baseline are 109.7 mL, 122.8 mL, and 0.917 L/sec, respectively, for changes in FEV_1_, FVC, and FEF_25–75_. These were calculated with a significance level of *p* = 0.0167 (0.05/3). Thus, for all dependent measures and the present sample size, relatively small changes were needed to detect a difference between exposure conditions.

## Conclusions

In conclusion, relatively brief, one-time exposures to mixtures of VOCs or VOCs and their oxidation products, at the upper bound of typical indoor concentration range, did not appear to cause significant acute changes in symptoms, neurobehavioral performance, or lung function in healthy women. In contrast, the psychological stressor was effective in producing increased autonomic arousal, as indicated by salivary cortisol, and in causing increased symptoms of anxiety, both well-documented effects of psychological stress. However, stress and exposure were neither synergistic nor additive in their effects on symptoms or neurobehavioral performance. The effect of stress, however, was isolated to symptoms of anxiety and did not generalize to other more typical symptoms associated with poor indoor air such as nasal irritation or headache. Although the irritation potency of complex and variable mixtures of VOCs and their oxidation products in buildings is difficult to predict, reported air concentrations of VOCs in buildings with poor indoor air quality are typically an order of magnitude lower than the VOC concentrations used in this study. Thus, our results suggest that the VOC concentration alone may not be the most salient factor to account for acute health complaints. Our present results support the conclusion that 3-hr exposures to VOCs or to the reaction products of VOCs and O_3_ at concentrations typically found in nonindustrial buildings are unlikely to be a significant cause of acute health complaints or effects for most occupants.

Several caveats need to be considered for our present study. This study included the largest number of subjects to date and intentionally selected only women for study because of their hypothesized vulnerability to report indoor air quality symptoms. However, the extent to which our results apply to indoor air problems experienced in a work environment with many other demands and chronic exposures is problematic. Thus, the lack of health effects observed may simply be a function of the necessarily acute exposure paradigm with healthy young subjects. Conversely, the exposure concentrations were quantitatively higher than those documented in most buildings with indoor air complaints. Furthermore, work demands were modeled in our study through use of a known stressor as well as requirements for computerized neurobehavioral tasks, and the former was successful in causing autonomic arousal and symptoms of anxiety. Another attribute of our study was that all exposure conditions were conducted at a relatively high air exchange rate (~ 1.8 air exchanges/hr), whereas health complaints associated with indoor air quality often occur in buildings with poor ventilation (i.e., air exchange rates were an order of magnitude lower than the air exchange rate used in our study). Overall, this study suggested that for a 2-hr time period, psychological stress may be a more potent factor than ambient chemical mixtures in the complaints attributed to poor indoor environments.

## Figures and Tables

**Table 1:** The mixture of VOCs.

No.	Compound	Relative weight	Concentration (mg/m^3^)
1	*n*-Butylacetate	10	8.25
2	*p*-Xylene	10	8.25
3	*n*-Butanol	1	0.825
4	*n*-Decane	1	0.825
5	1-Decene	1	0.825
6	1,1-Dichloroethane	1	0.825
7	*d*-Limonene	1	0.825
8	Ethylbenzene	1	0.825
9	Ethoxyethylacetate	1	0.825
10	*n*-Hexanal	1	0.825
11	*n*-Hexane	1	0.825
12	*n*-Nonane	1	0.825
13	α -Pinene	1	0.825
14	2-Butanone	0.1	0.083
15	Cyclohexane	0.1	0.083
16	3-Methyl-2-butanone	0.1	0.083
17	4-Methyl-2-pentanone	0.1	0.083
18	*n*-Pentanal	0.1	0.083
19	Isopropanol	0.1	0.083
20	*n*-Propylbenzene	0.1	0.083
21	1,2,4-Trimethylbenzene	0.1	0.083
22	*n*-Undecane	0.1	0.083
23	1-Octene	0.01	0.008
Total			26.330

**Table 2:** Summary of products observed during different exposure conditions.

Exposure conditions	Ultrafine particle number concentration (particles/cm^3^)^a^	Particle mass concentration (μg/m^3^)^b^	Formaldehyde (μg/m^3^)^b^	*p*-Tolualdehyde (μg/m^3^)^b^	Glyoxal (μg/m^3^)^b^	Hydrogen peroxide (ppb)^a,c^
MCA	NA^d^	< 5	7	ND	ND	NA^d^
VOCs (23 VOCs)	2,500	3–7	13	ND	ND	0.3
VOCs + O_3_ (O_3_ + 23 VOCs)	46,000	140	40	6.2	4.6	1.9

ND, not detected.

a Two-hour average concentration.

b Four-hour average concentration.

c Includes hydrogen peroxide and organic hydroperoxides.

d Not measured but expected to be the same or lower than the 23 VOC-only condition.

**Table 3:** Spirometry changes after exposures to MCA, VOCs, and VOCs + O_3_ (mean difference ± SD; *n* = 130).

	MCA	VOCs	VOCs ± O_3_	*p*-Value
Change in FEV_1_ (mL)	–50.46 ± 174.02	6.15 ± 176.56	–25.30 ± 210.33	0.05
Change in FVC (mL)	–71.54 ± 189.22	–42.62 ± 184.40	–60.38 ± 237.11	0.52
Change in FEF_25–75_ (L/sec)	–0.00 ± 0.41	0.51 ± 3.63	0.09 ± 0.53	0.12

Difference is the post-minus preexposure score. Bonferroni correction: *p* < 0.02.

**Table 4:** Effect sizes for symptom severity based on 130 subjects.

		VOC				Respiratory		
Change (from 15 min)	Total symptom^a^	General	Cognitive	Eye	Anxiety	Upper	Lower	Somatic
60 min	0.99	2.69	2.48	2.07	1.43	1.42	0.74	1.04
125 min	1.15	2.98	2.69	2.15	1.85	1.49	0.64	1.13

a Calculated at a significance level of 0.05. The effect sizes for all remaining subcategories of symptoms were calculated at a significance level of 0.05/7 = 0.0071.

## Appendix 1. Symptom list

**Table:**

VOC physical
Headache
Fatigue
Lightheaded
Drowsy
Nausea
VOC cognitive
Difficulty concentrating
Disoriented/confused
Dizzy
Eye irritation
Eye irritation (burning, dryness, or itching)
Runny/watery eyes
Anxiety
Feel jittery in body
Feel nervous
Heart palpitations
Feel tense
Worried
Upper respiratory
Sneeze
Nasal congestion
Choking
Throat irritation (burning or dryness)
Nose irritation, dryness, or itching
Lower respiratory
Short of breath
Wheezy
Chest tightening
Chest pain
Coughing
Somatic control
Skin irritation or dryness
Stomachache
Numbness/tingling
Ear ringing
Leg cramps
Back pain
Sweating
Body aches
